# Examining the Cellular Transport Pathway of Fusogenic Quantum Dots Conjugated With Tat Peptide

**DOI:** 10.3389/fbioe.2022.831379

**Published:** 2022-05-27

**Authors:** Jie Dai, Jun Wang, Xuan Yang, Zixing Xu, Gang Ruan

**Affiliations:** ^1^ Department of Biomedical Engineering, College of Engineering and Applied Sciences, Nanjing University, Nanjing, China; ^2^ Wisdom Lake Academy of Pharmacy, Xi’an Jiaotong-Liverpool University, Suzhou, China; ^3^ Nanobiotechnology and Nanomedicine Center, Xi’an Jiaotong-Liverpool University, Suzhou, China; ^4^ Shenzhen Research Institute of Nanjing University, Nanjing, China; ^5^ Institute of Materials Engineering, College of Engineering and Applied Sciences, Nanjing University, Nanjing, China

**Keywords:** nucleus entry, delivery, nanoparticle, quantum dot, vesicle escape, cell-penetrating peptide, targeting

## Abstract

Understanding the underlying transport mechanism of biological delivery is important for developing delivery technologies for pharmaceuticals, imaging agents, and nanomaterials. Recently reported by our group, SDots are a novel class of nanoparticle delivery systems with distinct biointerface features and excellent fusogenic capabilities (i.e., strong ability to interact with the hydrophobic portions of biomembranes). In this study, we investigate the cellular transport mechanism of SDots conjugated with Tat peptide (SDots-Tat) by live-cell spinning-disk confocal microscopy combined with molecular biology methods. Mechanistic studies were conducted on the following stages of cellular transport of SDots-Tat in HeLa cells: cellular entry, endosomal escape, nucleus entry, and intranuclear transport. A key finding is that, after escaping endosomes, SDots-Tat enter the cell nucleus via an importin *β*-independent pathway, bypassing the usual nucleus entry mechanism used by Tat. This finding implies a new approach to overcome the nucleus membrane barrier for designing biological delivery technologies.

## Introduction

Biological delivery is often critical for applications of pharmaceuticals, imaging agents, and nanomaterials (Kairdolf et al., 2013; Mitragotri et al., 2014; Mitchell et al., 2020; Souza et al., 2021; Tu et al., 2022). For developing biological delivery technologies, it is fundamental to understand the underlying biological transport mechanisms (Gilleron et al., 2013; Sahay et al., 2013; Lane et al., 2015; Chan, 2017; Finbloom et al., 2020). We recently reported a unique biological delivery concept termed ‘SDot,’ which demonstrated unprecedented ability of intracellular targeted delivery of quantum dots (QDs, i.e., semiconductor nanocrystals) (Wang et al., 2019). However, our understanding of the cellular transport mechanisms of SDots remains incomplete, and this lack of mechanistic understanding hinders further development of SDot-based delivery technologies. The present work seeks to gain a systematic understanding of the cellular transport pathway of SDots conjugated with Tat peptide (‘SDots-Tat’ for short), in which Tat peptide (Wadia et al., 2004; Ruan et al., 2007) has the ability of specific recognition of the cell nucleus in addition to the capacity to greatly enhance cellular uptake of the attached particles.

SDot is a new design of biointerface engineering and includes the following structural features: 1) naked hydrophobic nanoparticle surface, 2) a small portion of the nanoparticle surface is conjugated with a biofunctional molecule (e.g., Tat peptide in SDot-Tat), and 3) a solvent mixture containing a small percentage of minimally toxic organic cosolvent in addition to water (Wang et al., 2019). This structural design is distinct from that of conventional nanoparticles used for biological applications: the conventional nanoparticles have a predominantly hydrophilic interface with water to be used for biological applications. The rationale for the conventional design is that, because the biological environment is usually aqueous, the nanoparticle’s interface with the biological environment should be hydrophilic (water-loving) in order to be compatible with the biological environment. In contrast, the rationale for the SDot design is to mimic membrane proteins, which can use their large, nanometer-scale hydrophobic surface to penetrate into biological membranes without breaking the membranes (Wang et al., 2019). For SDot-Tat in particular, its interface with the biological environment is predominantly hydrophobic, with the Tat peptide (as the targeting ligand for the cell nucleus) taking only a small portion of the interface area; additionally, a small percentage of organic cosolvent with minimal toxicity is used to ensure dispersion of the nanoparticles in water. SDot is not intended to replace the conventional design in all biological applications; rather, SDot is only used in those cases where overcoming membrane barriers are important, given SDot’s superior membrane-penetrating ability (also known as fusogenic ability). In our recent study, SDots-Tat showed the extraordinary ability of intracellular-targeted delivery of quantum dots into the cell nucleus in live cells, crossing the following membrane barriers during the process: cell membrane, intracellular vesicle membrane, and nucleus membrane (Gilleron et al., 2013). Although SDots-Tat worked well as designed, a more detailed understanding of the transport mechanisms could offer new opportunities for further technology development.

In this study, we further investigate the biological mechanisms for the interactions of SDots-Tat with the aforementioned three membrane barriers. We seek to answer the following specific biological questions: 1) Do SDots-Tat use the usual mechanisms of Tat peptide to enter the cell and the cell nucleus? 2) At what specific stage do SDots-Tat escape intracellular endosomes? 3) Is the transport destination of SDots-Tat in the cell nucleus the same as Tat peptide?

## Experimental Methods

### Materials

Cadmium oxide (99.99%), zinc nitrate hexahydrate (98%), sulfur powder (99.98%), selenium powder (100 mesh, 99.5%), 1-octadecene (90%), stearic acid (95%), trioctylphosphine (90%), cysteamine (98%), 1-ethyl-3-(3-dimethylaminopropyl) carbodiimide (EDC), and N-hydroxysuccinimide (NHS) were purchased from Aldrich. N,N-dimethyl formamide (DMF, 99.5%) was purchased from Sinopharm Chemical Reagent. Tat peptide (sequence Ac-YGRKKRRQRRR) and Tat-TAMRA (sequence Ac-YGRKK(TAMRA)RRQRRR) were purchased from ChinaPeptides. The HeLa cell line was purchased from KeyGEN BioTECH. Dulbecco’s modified Eagle’s medium (DMEM), trypsin, phosphate buffer solution (PBS), and penicillin-streptomycin (PS) were purchased from Solarbio. Fetal bovine serum (FBS) was purchased from cellmaxBioTECH. Cell culture dishes and plates were purchased from NEST Biotech. Chondroitin sulfate, heparin, nystatin, dynasore, chlorpromazine hydrochloride, genistein, amiloride, cytochalasin D, and importazole were purchased from Shanghai BaiLi Biotechnology. Hoechst 33342 and Lipo3000 transfection reagent were purchased from Thermo Fisher Scientific.

### Preparation of SDots-Tat

SDots-Tat were synthesized by our recently reported method (Wang et al., 2019). Briefly, hydrophobic quantum dots were first synthesized by a scalable production method based on high-temperature crystallization without injection. Then, hydrophobic quantum dots (in DMF) were incubated with thioglycolic acid for 0.5 h to exchange a small percentage of the surface ligands of the quantum dots, so that the resulted quantum dots had –COOH on a small part of the quantum dot surface. The remaining hydrophobic surface coverage was estimated to be approximately 90%. The estimation was carried out with the quantity of the thioglycolic acid molecules added for ligand exchange, assuming all the thioglycolic acid molecules added had been attached onto the nanoparticle surface. The Tat peptide was conjugated with the –COOH group on the quantum dot surface using EDC chemistry (reaction addition ratio: one peptide molecule per nanoparticle). The solution was then dispersed in water (or cell culture medium), with a volume ratio of organic solvent to water 1:99, to form SDots-Tat.

### Live-Cell Spinning-Disk Confocal Microscopy

HeLa cells were cultured in DMEM supplemented with 10% FBS at 37°C with 5% CO_2_ and 1% PS. Live-cell imaging studies were performed using a live-cell spinning-disk confocal microscopy system consisting of a cell incubation chamber (IX3W, Tokai Hit), an epi-fluorescent microscope (IX-83, Olympus), a spinning-disk confocal system (Andor), and an electron-multiplying charge-coupled device (EMCCD) camera (Evolve 512, Photometrics). The cells were first seeded at ∼30% confluency on a glass-bottomed cell culture dish (0.17 mm thickness for the glass bottom). Following a 12-h incubation period at the conditions of 37°C and 5% CO_2_, the cell culture medium was replaced by dispersion of SDots-Tat (1 nM nanoparticles in total solvent volume, 1% DMF as the organic cosolvent in aqueous cell culture medium, 90% hydrophobic surface coverage on nanoparticle, one Tat peptide per nanoparticle, and fluorescent emission peak 545 nm). After incubation with SDots-Tat for a specific time duration (e.g., 15 min, 1, 4, 6, 8, 12, and 24 h), the cells were washed with PBS three times to remove SDots-Tat outside the cells or loosely bound to the outer cell surface, and the cells were imaged using the live-cell spinning-disk confocal microscopy system. To counter-stain the cell nucleus, right before imaging (at a particular time point of cellular transport), the fluorescent dye Hoechst 33342 (blue fluorescent color, 5 µM in cell culture medium) was incubated with live cells for 20 min. Image processing and analysis were conducted using MetaMorph and ImageJ software.

### Plasmid Construction and Transfection

The proteins Rab5, Rab7, and B23 were labeled by the red fluorescent protein mCherry in live cells by plasmid transfection. The plasmid vector was pcDNA3.1 (+). The target sequences of Rab5, Rab7, and B23 were selected from the NCBI gene database. The recombinant plasmids were purchased from ChinaPeptides. Plasmid transfection was performed using the Lipo3000 transfection reagent.

## Results and Discussion

### Examining the Cellular Entry of SDots-Tat

SDots-Tat with QDs as the nanoparticle core material were prepared as we previously described (Wang et al., 2019). The hydrodynamic diameter of SDots-Tat was ∼7 nm as measured by dynamic light scattering. This is much smaller than the size of the nuclear pore complex core (diameter 110 nm and height 70 nm) (Knockenhauer and Schwartz, 2016). The surface charge of SDots-Tat was ∼13 mV as measured by zeta potential. The bright and stable fluorescence of QD (Ruan et al., 2007) permitted us to use a live cell spinning-disk confocal microscopy system to visualize the cellular transport process of SDots-Tat. HeLa cells were used in this work. The minimal cytotoxicity of SDots-Tat was verified by MTT assay (Wang et al., 2019).

**FIGURE 1:**
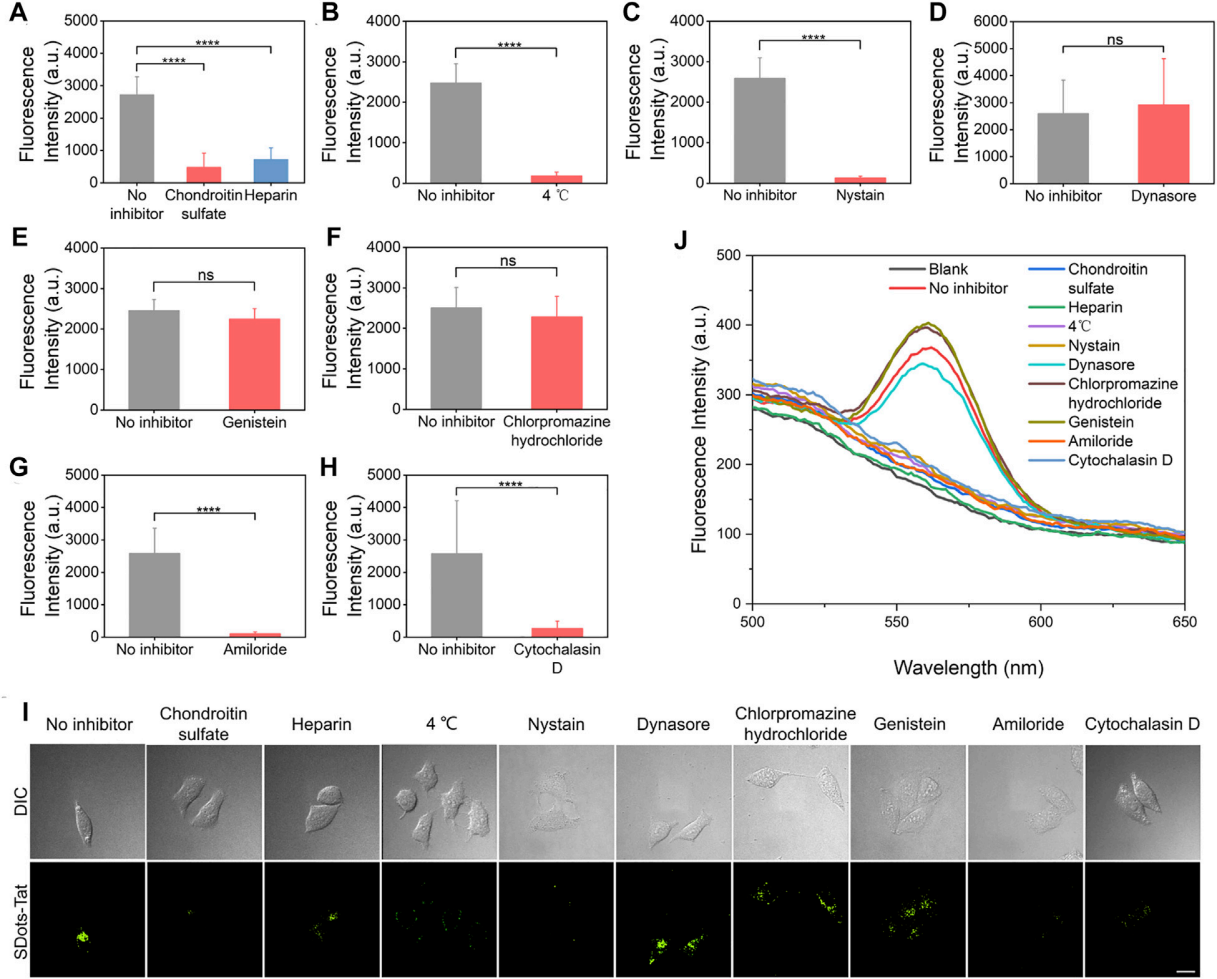
Inhibitor studies of the cellular entry process of SDots-Tat. **(A–H)** Quantification results based on live-cell fluorescent microscopy. Error bars, mean ± s.e.m, *n* >50. ***, *p* < 0.001; ns, not significant (Student’s t-test). The cells were pretreated with inhibitors for 30 min and were then incubated with SDots-Tat for 30 min. **(A)** Inhibitors for proteoglycans; chondroitin sulfate, 50 μg/ml; heparin, 25 μg/ml. **(B)** Low-temperature inhibition of endocytosis. **(C)** Inhibitor for lipid raft, nystatin, 25 μg/ml. **(D)** Inhibitor for dynamin, dynasore, 10 μM. **(E)** Inhibitor of clathrin-mediated endocytosis, chlorpromazine hydrochloride, 100 μg/ml. **(F)** Inhibitor for caveolae-mediated endocytosis, genistein, 17.5 μg/ml. **(G)** Inhibitor of macropinocytosis, amiloride, 100 μM. **(H)** Inhibitor of F-actin, cytochalasin D, 2 μM. **(I)** Corresponding representative confocal fluorescent microscopy images. Scale bar, 20 μm. **(J)** Results of inhibitor studies analyzed by an alternative method: the cells were removed from the cell culture plates 2 h after incubating SDots-Tat with the cells (the cells were pretreated with the inhibitors for 30 min). The incubation time with the cells was selected to balance two considerations: 1) if the incubation time was too short, we would not be able to detect significant cellular uptake amount for all samples; 2) if the incubation time was too long, the cells could develop ways to recover from the inhibitor effects so that we would not be able to distinguish the differences in cellular uptake between different samples. Subsequently, 5 × 10^6^ cells were broken by probe sonication, and fluorescent spectroscopy was used to analyze the quantity of SDots-Tat uptake. “Blank” refers to the result with no SDots-Tat being incubated with the cells.

A series of inhibitor studies were performed to investigate the specific cellular entry mechanism(s) of SDots-Tat. To examine the binding of SDots-Tat with the cell membrane, two inhibitors (i.e., chondroitin sulfate and heparin) for binding with cell surface proteoglycans were used (Baker, 2010; Silhol et al., 2002). Significant reduction in cellular uptake of SDots-Tat was found with the use of either of these two inhibitors (Figure 1A, *p* < 0.001), indicating that proteoglycans functioned as the cell surface receptors for SDots-Tat. The nature of the binding between SDots-Tat and proteoglycans was presumably electrostatic attraction between the many positive charges in the Tat molecules and the many negative charges in the proteoglycan molecules (Tyagi et al., 2001; Silhol et al., 2002).

After binding with the cell membrane, the experimental results suggest that SDots-Tat entered the cells primarily *via* endocytosis, while a small portion of the nanoparticles could penetrate through the cell membrane *via* an energy-independent process. As shown in Figure 1B, after using low temperature (4°C) to block endocytosis, approximately 7% of the nanoparticles (compared to those at 37°C) could still enter the cells. We then investigated which of the known endocytosis process(es), including clathrin-mediated endocytosis, caveolae-mediated endocytosis, and macropinocytosis, was used by SDots-Tat. Phagocytosis was excluded in the study since this endocytosis process only occurs in the highly specialized cell types known as phagocytes.

Lipid raft is known for its involvement in caveolae-mediated endocytosis and macropinocytosis. Nystatin, an inhibitor for lipid raft (Liu et al., 2002), yielded a significant decrease in the cellular uptake of SDots-Tat (Figure 1C, *p* < 0.001), indicating the involvement of lipid raft in SDots-Tat’s endocytosis. Dynamin is required in clathrin-mediated endocytosis and caveolae-mediated endocytosis (Henley et al., 1999). Dynasore, an inhibitor for dynamin’s GTPase activity (Macia et al., 2006), did not result in a significant change to the cellular uptake of SDots-Tat (Figure 1D), indicating the noninvolvement of dynamin in SDots-Tat’s endocytosis. To further exclude the possible involvement of clathrin-mediated endocytosis in SDots-Tat’s cellular uptake, chlorpromazine hydrochloride, an inhibitor of clathrin-mediated endocytosis (Wu et al., 2019), was used. It was found that this inhibitor failed to significantly change the quantity of SDots-Tat’s cellular uptake (Figure 1E), thus confirming the noninvolvement of clathrin-mediated endocytosis. Subsequently, we blocked caveolae-mediated endocytosis with the inhibitor genistein (Parton et al., 1994), and the cellular uptake of SDots-Tat was not significantly reduced (Figure 1F). This indicates that caveolae-mediated endocytosis was not involved. We then blocked macropinocytosis with the inhibitor amiloride (West et al., 1989), which could block the Na^+^/H^+^ exchange required in macropinocytosis. The experimental results showed that the cellular uptake of SDot-Tat was nearly completely blocked (Figure 1G, *p* < 0.001). This result suggests the involvement of macropinocytosis. Finally, the F-actin inhibitor cytochalasin (Muriel et al., 2017) led to a significant decrease in SDots-Tat’s cellular uptake (Figure 1H, *p* < 0.001), indicating the involvement of F-actin.

In addition to live-cell microscopy, we also employed an alternative method to analyze the inhibitor results for the cellular uptake mechanism. After the respective inhibitor treatment and SDots-Tat incubation with the cells, we removed the cells from the cell culture plates and disrupted the cells with probe sonication. After removing the debris by centrifugation, fluorescent spectroscopy was utilized on the remaining dispersion to analyze the cellular uptake of SDots-Tat. As shown in Figure 1J, the trend of the results is the same as that analyzed by live-cell microscopy.

Taken together, when SDots-Tat encountered the cells, the nanoparticles first bound with proteoglycans on the cell surface, then were internalized by lipid raft-dependent, F-actin-dependent macropinocytosis. Additionally, a small portion of the nanoparticles could also enter the cells via energy-independent penetration through the cell membrane. Previously, it has been reported that Tat peptide-conjugated water-soluble QDs are internalized by HeLa cells only *via* macropinocytosis (Ruan et al., 2007). Thus, the only difference in cellular uptake mechanism between SDots-Tat and Tat peptide-conjugated water-soluble QDs is that, in addition to macropinocytosis, the former also uses energy-independent membrane penetration, presumably due to the strong fusogenic ability of SDots-Tat.

### Examining the Endosomal Escape of SDot-Tat

Colocalization studies were used to investigate the endosomal escape process of SDots-Tat after they entered the cells. Specifically, we sought to examine the timeline of the endosomal escape process. In addition to examining how fast the process was, we were interested in the roles of two specific types of endosomes, namely, early endosomes and late endosomes. Rab5 and Rab7 are proteins specifically located in early endosomes and late endosomes, respectively (Chavrier et al., 1990; Nielsen et al., 1999). In two separate sets of live cells, we labeled Rab5 and Rab7 with the red fluorescent protein mCherry by plasmid transfection, respectively. We then incubated the two separate sets of cells with SDots-Tat (green fluorescence), to study the colocalization of SDots-Tat with early endosomes and late endosomes, respectively.

**FIGURE 2:**
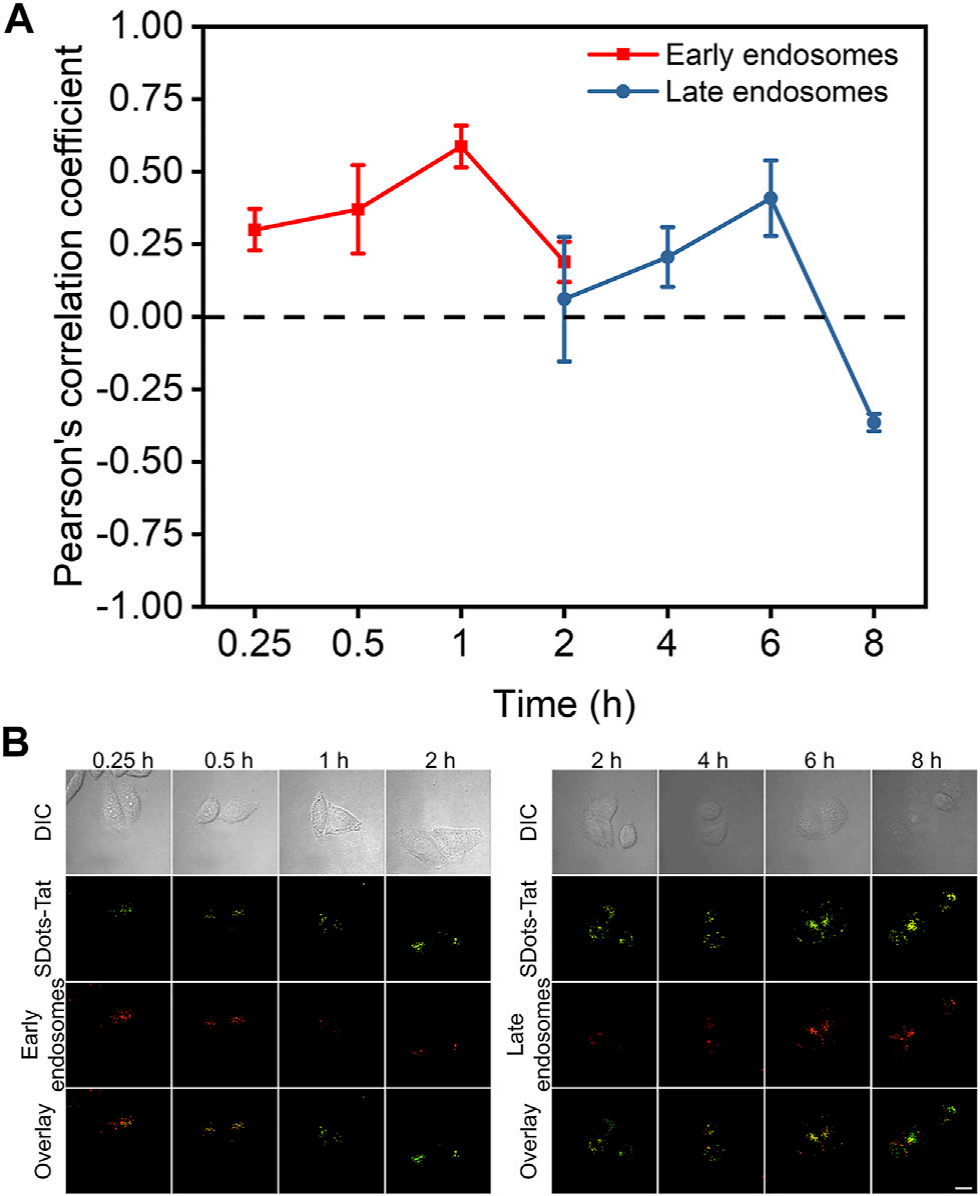
Colocalization studies of SDots-Tat with two specific types of endosomes, i.e., early endosomes and late endosomes. **(A)** Quantification of the degree of colocalization by Pearson’s correlation coefficient (PCC) analysis based on confocal fluorescent images. The PCC values range between -1 and 1. A larger PCC value indicates a greater degree of colocalization. PCC = 1 corresponds to perfect correlation (colocalization); PCC =-1 corresponds to anticorrelation (no colocalization). Error bars, mean ± s.e.m, *n* >50. **(B)** Representative confocal fluorescent images for the colocalization studies. SDots-Tat show green fluorescence. Early endosomes (Rab5) and late endosomes (Rab7) show red fluorescence. Scale bar, 20 μm.

As shown in Figure 2, the changes in the degree of colocalization (quantified by Pearson’s correlation coefficient) between SDots-Tat and either of the two different types of endosomes went through a similar trend, i.e., an increase to a maximum degree of colocalization followed by a decrease to a low degree of colocalization. Interestingly, there was a delay in the colocalization kinetics curve for late endosomes compared to that for early endosomes (Figure 2A). The decrease in colocalization for early endosomes nearly coincided with the increase in colocalization for late endosomes (Figure 2A). These results suggest that, upon the cellular entry of SDots-Tat, they were first trapped in early endosomes and were then gradually transferred to late endosomes. Transferring of SDots-Tat from early endosomes to late endosomes occurred between 1 and 6 h (incubation time of SDots-Tat with the cells) (Figure 2A). By 8 h, a majority of SDots-Tat had escaped from late endosomes (Figure 2A).

### Examining the Nucleus Entry of SDots-Tat

**FIGURE 3:**
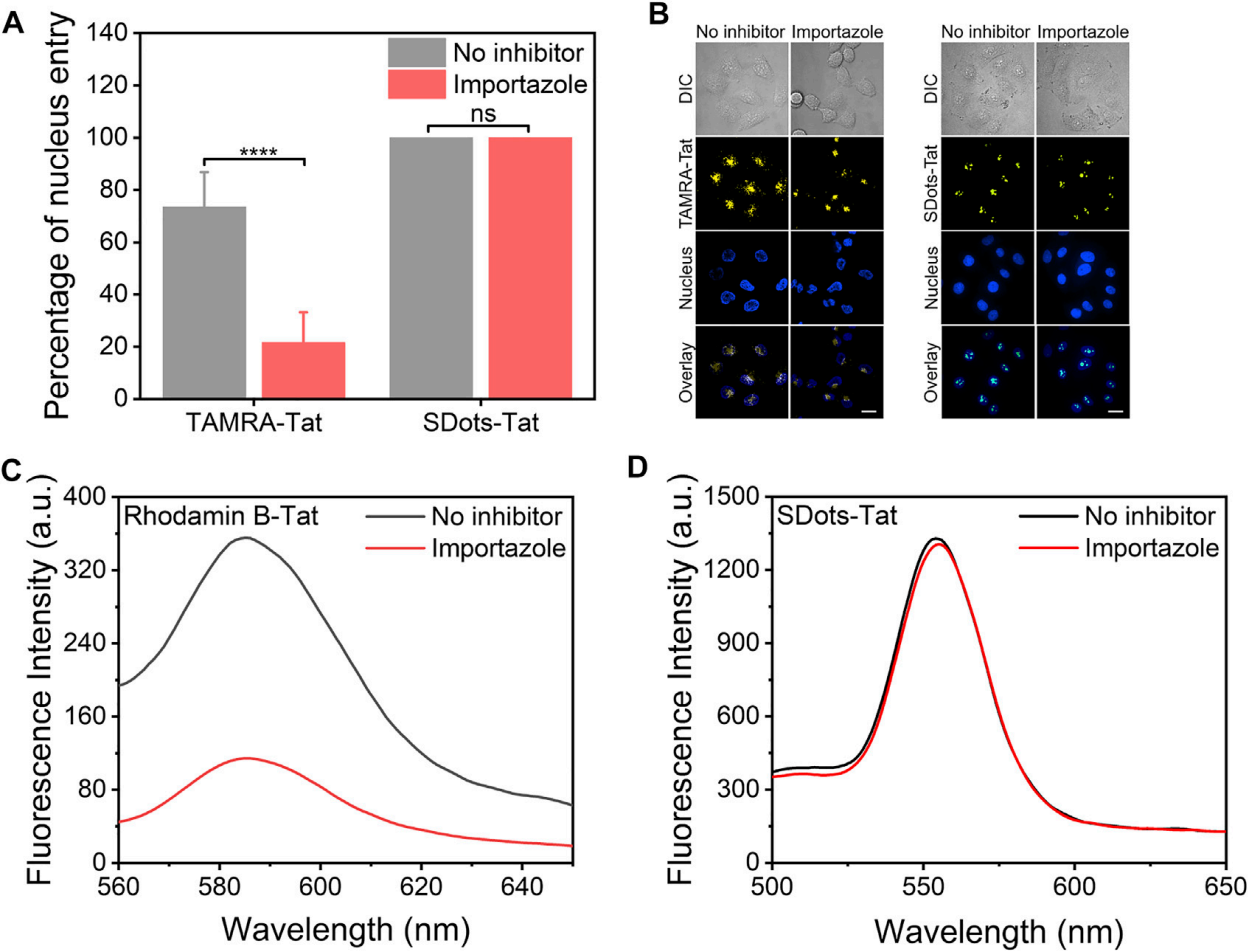
Nucleus entry mechanism of SDots-Tat. Importazole, 40 μM, 12 h preincubation with the cells before the addition of SDots-Tat. **(A)** Quantification of the percentage of nucleus entry based on live-cell confocal fluorescent images. Error bars, mean ± s.e.m, *n* >50. ****, *p* < 0.0001; ns, not significant (Student’s t-test). **(B)** Representative live-cell confocal fluorescent images for the quantification of nucleus entry. Hoechst 33342 (2 μg/ml) was used to label the cell nucleus. Scale bar, 20 μm. **(C,D)** Alternative method used to analyze the nucleus entry of dye-Tat or SDot-Tat. The cell nuclei were isolated from the cells before fluorescent spectrum measurement. **(C)** Rhodamin B-Tat. **(D)** SDot-Tat.

It has been previously reported that the Tat peptide enters the cell nucleus via importin *β*, a nuclear transport receptor (Cautain et al., 2015). Here, a striking finding was that SDots-Tat did *not* use importin *β* to enter the cell nucleus. The importin *β* inhibitor importazole was used, which can block the function of importin *β* by disrupting the binding of RanGTP with importin *β* (Jans et al., 2019). We employed two different methods to characterize the nucleus entry, and they both showed the same trend. First, we utilized live-cell microscopy to study the nucleus entry and found that the use of importazole had virtually no effect on the amount of SDots-Tat entering the cell nucleus, while importazole greatly reduced the nucleus entry amount of TAMRA-Tat (TAMRA is a small molecule fluorescent dye, Figures 3A, B). Second, we isolated the cell nucleus for 12 h after incubating the cells with SDots-Tat or rhodamin B-Tat (rhodamin B is a small molecule fluorescent dye). The nucleus entry of SDots-Tat or rhodamin B-Tat was analyzed by examining the fluorescent spectra of the dispersion formed by the aforementioned isolated nucleus. As shown in Figures 3C,D, this analytical method yields the same trend of the results: importazole substantially blocked the nucleus entry of rhodamin B-Tat but failed to inhibit the nucleus entry of SDots-Tat.

### Examining the Intra-nuclear Fate of SDots-Tat

**FIGURE 4:**
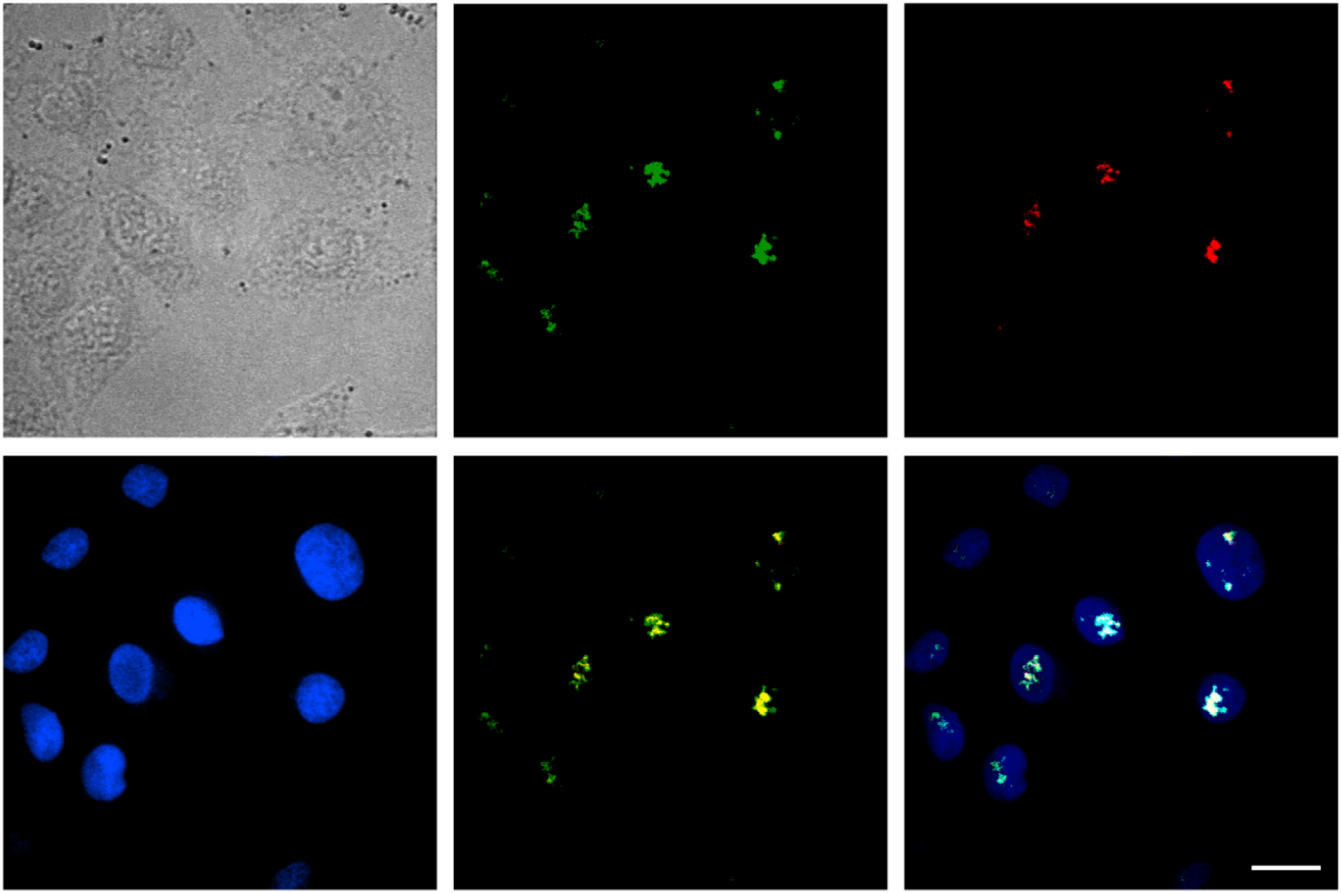
Intranuclear fate of SDots-Tat. The top left image is a bright-field image of cells. The top middle image is for SDots-Tat (green fluorescence). The top right image is for the B23 protein located in the nucleolus (red fluorescence). The bottom left image is for the cell nucleus (labeled by Hoechst 33342, 2 μg/ml). The bottom middle image is an overlay of SDot-Tat and B23 protein. The bottom right image is an overlay of SDot, B23 protein, and nucleus. Scale bar, 20 μm.

Inside the cell nucleus, the basic structural components include chromatin, nucleolus, nucleus matrix, and nucleus scaffold. It has been previously reported that, in the cell nucleus, Tat protein is specifically bound with B23 protein, which is located in the nucleolus (Dang and Lee, 1989; Li, 1997). Here, we examined whether the specific binding with B23 protein occurred for SDots-Tat. We labeled B23 protein with the red fluorescent protein mCherry in live cells by plasmid transfection. As shown in Figure 4, after entering the cell nucleus, SDots-Tat showed a high degree of colocalization with B23 protein (Pearson’s correlation coefficient 0.72 ± 0.08, determined for >50 cells), indicating that SDots-Tat’s fate in the cell nucleus is forming a complex with B23 protein in the nucleolus. Control images are shown in Supplementary Figure S1 to confirm that there is no leakage of fluorescent signal from the red channel to the green channel. In addition, Supplementary Videos S1,2 show three-dimensional reconstruction confocal images, confirming colocalization of SDots-Tat with the cell nucleus.

### Cellular Transport Pathway of SDots-Tat

**FIGURE 5:**
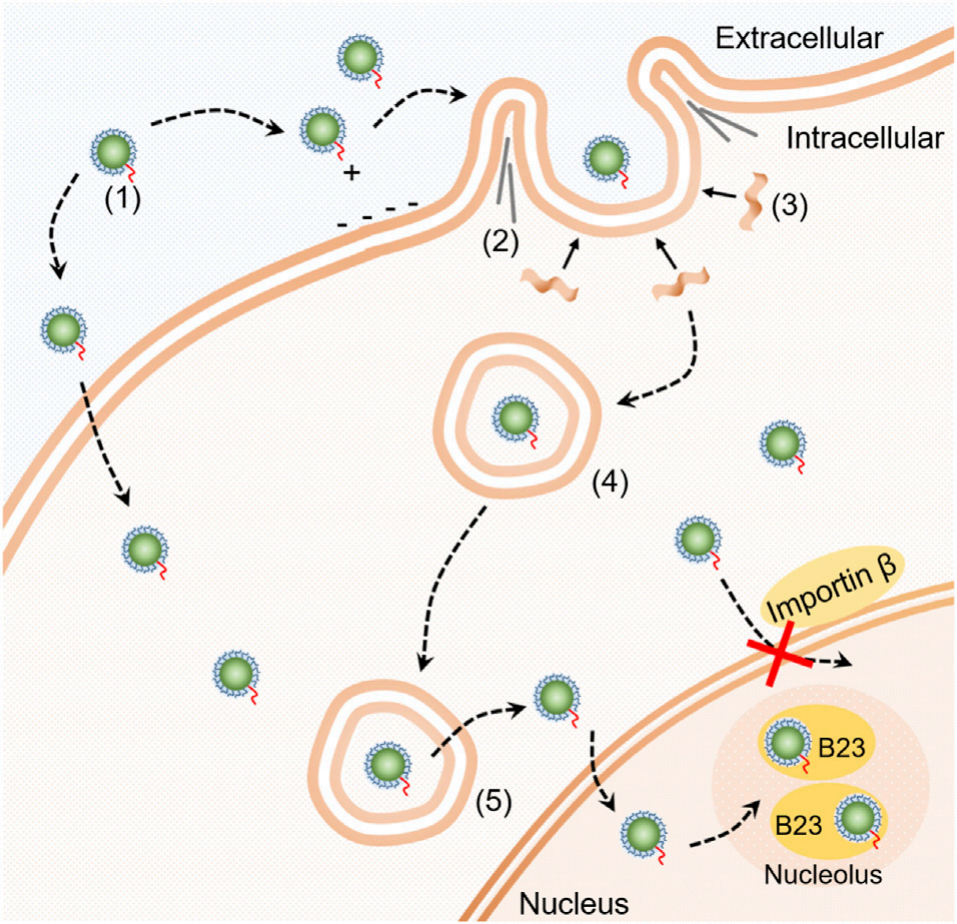
Schematic diagram of the cellular transport pathway of SDot-Tat. (1) SDot-Tat. (2) F-actin. (3) Lipid raft. (4) Early endosome. (5) Late endosome.

Figure 5 shows a schematic summarizing of our findings on the cellular transport pathway of SDots-Tat. SDots-Tat bind with proteoglycans on the cell surface via electrostatic attraction. This is followed by the cellular entry of SDots-Tat via lipid raft-dependent, F-actin-dependent macropinocytosis. A parallel, yet lesser in internalization quantity, cellular entry route of SDots-Tat is energy-independent penetration of the cell membrane. For those SDots-Tat entering the cells through macropinocytosis, they are initially trapped in endosomes. They are gradually transferred from early endosomes to late endosomes, and all of them escape from these endosomes within ∼8 h. Subsequently, SDots-Tat enter the cell nucleus *via* an importin *β*-independent mechanism. Finally, after entering the cell nucleus, SDots-Tat bind with B23 protein in the nucleolus.

These results suggest that the presence of SDot could alter the mechanisms of some steps in the cellular transport pathway of Tat. Most remarkably, with SDot, the nucleus entry mechanism of Tat was changed from importin *β*-dependent to importin *β*-independent. The exact mechanism of SDot-Tat’s nucleus entry is unclear at this point. A possible reason for the change in the nucleus entry mechanism is that the structural features of SDot (e.g., hydrophobic surface and cosolvent) could yield enhanced interaction with the nucleus membrane, and thus SDot-Tat could cross the nucleus membrane without the help of importin *β*. The finding that SDot can lead to an alternative nucleus entry mechanism has important implications for developing biological delivery technologies. The action sites of many pharmaceuticals are inside the cell nucleus. For the applications of these pharmaceuticals, nucleus entry is frequently a critical challenge (Ding et al., 2017). Our finding on nucleus entry suggests that an alternative nucleus entry mechanism could be used by these pharmaceuticals if they are delivered by a delivery system with the structural features of SDot. It should be noted that, in the present study, only one type of core material (quantum dots) was used to examine the biological behaviors of SDots. Thus, further studies are needed to examine other core materials (e.g., polymers and iron oxides).

## Conclusion

We have performed a systematic investigation on the biological pathway of SDot-Tat’s cellular transport. The findings highlight SDot’s ability to facilitate biological transport, including altering the biological mechanisms of crossing certain transport barriers. The insights gained would be helpful for designing delivery systems to overcome transport barriers in the biological world.

## Data Availability

The raw data supporting the conclusion of this article will be made available by the authors, without undue reservation.
