# Supplementary material for: Examining the Cellular Transport Pathway of Fusogenic Quantum Dots Conjugated With Tat Peptide
Source: Front Bioeng Biotechnol. 2022 May 27;10:831379. doi: 10.3389/fbioe.2022.831379 (PMC9184739; doi:10.3389/fbioe.2022.831379)
Supplement: Supplementary file 2 [file Presentation1.pdf]

SUPPLEMENTARY INFORMATION

Examining the Cellular Transport Pathway of Fusogenic Quantum Dots Conjugated with Tat Peptide

Jie Dai [1], Jun Wang[1], Xuan Yang [2,3], Zixing Xu[1], Gang Ruan [1, 2, 3, 4, 5,*]

[1] Department of Biomedical Engineering, College of Engineering and Applied Sciences, Nanjing University, China
[2] Wisdom Lake Academy of Pharmacy, Xi'an Jiaotong-Liverpool University, Suzhou, China
[3] Nanobiotechnology & Nanomedicine Center, Xi'an Jiaotong-Liverpool University, Suzhou, China
[4] Shenzhen Research Institute of Nanjing University, China
[5] Institute of Materials Engineering, College of Engineering and Applied Sciences, Nanjing University, China

* Corresponding Author Gang.Ruan@xjtlu.edu.cn

This Supplementary Information file contains the following:

Supplementary Figures 1.

Descriptions of Supplementary Videos 1-2.

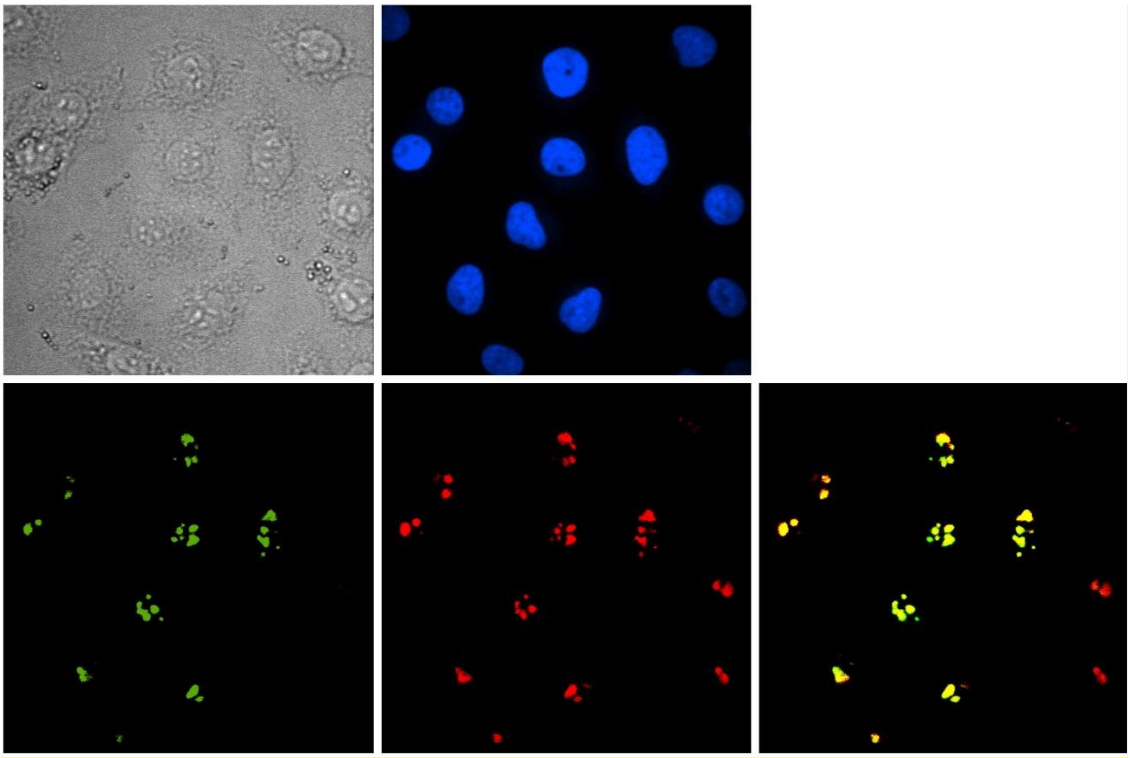

Supplementary Figure 1 Control images for studying the intra-nuclear fate of SDots-Tat with colocalization. These control images show that the red channel signal does NOT leak to the green channel. The red channel image is the bottom-middle image; the green channel image is the bottom-left image. As can be seen, in the red channel image there are some fluorescent spots that are NOT seen in the green channel image. This observation demonstrates that there is NO leaking of signal from the red channel to the green channel.

Supplementary Videos

Supplementary Video 1 Three dimensional reconstruction confocal images to show colocalization of SDots-Tat (green) with cell nucleus (blue, stained by the nucleus dye Hoechst 33342). Colocalization of the cell nucleus and cS-bQDs-Tat leads to the composite color watchet blue. 60× objective was used to capture the images.

Supplementary Video 2 Three dimensional reconstruction confocal images to show colocalization of SDots-Tat (green) with cell nucleus (red, stained by the nucleus dye Hoechst 33342). Colocalization of the cell nucleus and cS-bQDs-Tat leads to the composite color yellow. 20× objective was used to capture the images. More cells are shown in the view than Supplementary Video 1.
